# Silver-Exchanged Clinoptilolite-Rich Natural Zeolite for Radon Removal from Air

**DOI:** 10.3390/ma18071465

**Published:** 2025-03-26

**Authors:** Marin Senila, Oana Cadar, Robert-Csaba Begy, Claudiu Tanaselia, Dorina Simedru, Cecilia Roman

**Affiliations:** 1 INCDO-INOE 2000, Research Institute for Analytical Instrumentation, 67 Donath Street, 400293 Cluj-Napoca, Romania; oana.cadar@icia.ro (O.C.); claudiu.tanaselia@icia.ro (C.T.); dorina.simedru@icia.ro (D.S.); cici_roman@yahoo.com (C.R.); 2 Institute for Interdisciplinary Research in Bio-Nano-Sciences, Babes-Bolyai University, Treboniu Laurian 42, 400271 Cluj-Napoca, Romania

**Keywords:** clinoptilolite, silver exchanged zeolite, radon removal, zeolite modification, indoor air, adsorption

## Abstract

Radon (Rn) is a hazardous radioactive gas that poses significant health risks in enclosed indoor environments. This study investigates the potential of silver-exchanged clinoptilolite-rich natural zeolite (NZ-Ag^+^) for the removal of Rn from air. Natural zeolite (NZ) was thermally treated and further modified to enhance its adsorption characteristics. The thermally treated NZ (200 °C) was first exchanged in Na^+^ form, since Na^+^ is more easily exchanged in clinoptilolite with hydrated Ag^+^ ions than the other exchangeable cations. The modification with Ag^+^ was carried out at room temperature using ultrasonic processing to obtain (NZ-Ag^+^). The materials were characterized in terms of chemical composition, cation exchange capacity, mineralogy, total surface area, pore volume, and thermal behavior. Rn adsorption experiments were performed using a closed-circuit system, and the efficiency of NZ-Ag^+^ was compared with that of NZ. The results indicate that NZ-Ag^+^ exhibits superior Rn adsorption capacity, achieving up to 50% higher retention efficiency compared to NZ. The improved performance is attributed to enhanced adsorption facilitated by silver ion clusters interacting with radon atoms. These results suggest that silver-exchanged zeolite represents a promising material for radon mitigation in air filtration systems, with potential applications in residential and occupational settings.

## 1. Introduction

Radon (Rn) gas is a ubiquitous contaminant that belongs to the natural radioactive decay series of ^238^U, which occurs naturally in the Earth’s crust. This noble gas is odorless, colorless, and tasteless [1]. Exposure to radioactive disintegration products of the chemically inert gas ^222^Rn accounts for about half of all non-medical exposures to ionizing radiation [2]. The adverse effects of ^222^Rn are mostly attributed to its short-lived alpha-emitting decay products (^218^Po and ^214^Po), which are solid and, if inhaled, can be deposited in the respiratory tract and expose cells to radiation [3]. Therefore, long-term exposure to levels exceeding 100 Bq/m^3^ is considered a serious environmental and health problem [4]. Normally, Rn concentrations in the outdoor air are low but may increase indoors, resulting in exposure of occupants. After smoking, Rn represents the second leading cause of lung cancer worldwide [5].

The Directive 2013/59/EURATOM establishes action plans and limit values for Rn exposure in buildings and workplaces with the aim of minimizing human Rn inhalation. Considering these aspects, Rn has attracted considerable research interest [6,7,8]. Indoor air pollution by Rn can be controlled by using strategies such as building site and material selection, Rn-resistant construction, and ventilation [9]. In existing buildings, Rn increases in indoor air due to improperly functioning ventilation systems. In general, the existing ventilation systems include ducts, particle filters, air conditioners, and fans that cannot remove Rn from the air [10]. A possibility to reduce the Rn content in the air is to use adsorbent materials in the filter composition.

As a noble gas, Rn is difficult to ionize, so its retention is based on physisorption due to the van der Waals forces rather than the formations of strong chemical bonds [8]. Thus, its retention is enhanced in porous materials with increased specific surface area. Activated carbon has been studied as an adsorbent material for Rn removal [11], but research on other porous materials capable of adsorbing Rn from air is very scarce.

Natural zeolites are widely available and are inexpensive minerals with porous crystalline structures and well-defined channels or cavities [12,13]. They have open-framework aluminosilicates with negative charges that are compensated by extra-framework cations (typically alkali or alkaline earth metal cations) [14]. Zeolites can be tailored for different applications, making them suitable materials for Rn adsorption. Clinoptilolite is one of the most abundant natural zeolites mined in many countries and has a good sorption due to its physicochemical characteristics. In addition to the microporous structure, which provides its ability to act as a molecular sieve and to adsorb molecules of appropriate cross-sectional diameter, clinoptilolite has negative charge, which provides its ion exchange capacity [15,16].

The removal of radionuclides from various environmental compartments using zeolites is an accepted practice [17]. In this regard, the clinoptilolite type zeolite, due to its ion exchange characteristics, is a well-known example in the mitigation of radioactive isotopes released at the Chernobyl nuclear accident [18]. When the substance to be removed is an inert gas such as Rn, the removal mechanism may not be possible due to the excellent ion exchange characteristics of zeolites. Therefore, the scientific literature addressing the efficiency of natural and synthetic zeolites for the removal of Rn is scarce. Among the published papers, very few refer to the use of natural zeolites for Rn removal from air. Bikit et al. [19] tested natural zeolites of different granulation for Rn adsorption from air in a closed chamber. They reported that natural zeolite has a lower adsorption coefficient of Rn (0.038 m^3^kg^−1^–0.11 m^3^kg^−1^) compared to activated carbon and that the grain size of zeolite does not strongly influence its adsorption coefficient. Osmanlioglu [20] reported 85% removal of the radioactive Rn gas by using natural zeolite as a filter in a closed system. Kang et al. [8] used fluorine-functionalized natural zeolite to remove Rn from underground water, while He et al. used natural zeolite as an additive to reduce radioactive emission from fly ash-containing building materials [21].

Several studies on Rn capture have been carried out using synthetic zeolites and silver-exchanged synthetic zeolites [22,23,24,25,26]. Paschalides et al. [23] used synthetic zeolite 5A with a pore size of 0.5 nm as a passive Rn collector to improve the lower limit of detection (LLD) in a Rn measurement system. The LLD for the synthetic zeolite system was about 45 Bq/m^3^, similar to that of activated carbon and much lower than that of blue silica gel (~350 Bq/m^3^), indicating a better adsorption efficiency of the zeolite. Studies on silver exchanged-synthetic zeolites have shown that they are more efficient for the retention of noble gases than their sodium forms [22,24,27,28]. Hedström et al. [24] stated that Ag-exchanged synthetic zeolite 13X exhibited an excellent efficiency (~90%) for the removal of Rn from a closed system. Heinitz et al. [25] reported remarkable adsorption coefficients for Rn in the air, greater than 3000 m^3^/kg at 293 K, for synthetic silver-exchanged zeolites (Ag-ETS-10 and Ag-ZSM-5).

The larger volume of the Rn atom and its lower ionization energy in the group of noble gases in the periodic table make this element more susceptible than other noble gases to donate an electron [29], which creates the premises for increasing the efficiency in Rn gas capture. Studies on silver-exchanged zeolites have also revealed that their bactericidal, antifungal, antiseptic, and germicidal characteristics provide additional benefits in using this material in air filtration systems [30,31,32,33,34].

Since, to the best of our knowledge, this has not been previously studied, this paper aims to investigate the adsorption of Rn from air filtered through natural clinoptilolite-type zeolite and silver-exchanged clinoptilolite-type zeolite, in order to test the hypothesis that the silver ions loaded on the natural zeolite increase the efficiency of Rn removal from contaminated air. The experiments were carried out in a specially designed closed circuit consisting of a Rn generator, a Rn tank, a measuring unit, an adsorbent sample cell into which the adsorbent material to be tested is introduced, and a desiccator.

## 2. Materials and Methods

### 2.1. Chemicals

All reagents used in the experiments were at least of analytical purity. Emsure^®^ ACS premium grade acids HNO_3_ 65%, HCl 37%, and HF 40% were purchased from Merck (Darmstadt, Germany). Ultrapure water (18 MΩ cm^−1^) obtained from a Millipore Direct Q3 (Millipore, Molsheim, France) was used for dilutions. Multielement standard solution IV for ICP (CertiPUR IV, traceable to NIST, Merck, Darmstadt, Germany) containing 23 elements 1000 mg L^−1^, purchased from Merck (Darmstadt, Germany) was used to prepare calibration standards in the range 0–20 mg L^−1^ element by serial dilution with 2% (*v*/*v*) nitric acid. The accuracy of the analysis for total metal concentrations in zeolite was assessed using CRM BCS-CRM 375/1 soda feldspar (Bureau of Analyzed Samples, Middlesbrough, UK). The percent recoveries of the elements analyzed in the CRM ranged from 92 to 110%.

### 2.2. Natural and Silver-Exchanged Zeolite Characterization

Natural zeolite (NZ) material with particle sizes of 0.5–1.0 mm was obtained from the Zeolites Production factory, which exploits a quarry located in Racos, Brasov County, Romania. In the laboratory, the zeolite was thermally treated at a temperature of 200 °C for 4 h to eliminate water and gases that could block the microchannels.

Microwave-assisted acid digestion of zeolite samples with a mixture of concentrated HNO_3_:HCl:HF (3:9:2, *v*:*v*:*v*) using an Xpert system (Berghof, Eningen, Germany) was used for the determination of major elements (Al, Ca, Mg, K, Na, Fe, Mn). A three-step digestion program was applied: (1) 10 min at 200 °C, (2) 30 min at 220 °C, and (3) 15 min at 100 °C. After cooling down, 2 g of H_3_BO_3_ were added, and the mixtures were heated again at 220 °C for 30 min. The resulting solutions were filtered and diluted to 100 mL with ultrapure water. The determination of Al, Ca, Mg, K, Na, Fe, Mn, and Ag was performed by ICP-OES using an Optima 5300 DV spectrometer (Perkin Elmer, Waltham, MA, USA). The oxide content of each major element in the total sample was then calculated using the atomic and molecular masses. Loss of ignition (LOI) and SiO_2_ were determined gravimetrically [34]. Cation exchange capacity (CEC) was determined by measuring the major cations (K^+^, Na^+^, Ca^2+^, and Mg^2+^) exchanged in ammonium acetate solution, according to a method adapted from US EPA Method 9080 [35].

XRD measurements were performed using a Bruker D8 Advance (Bruker, Karlsruhe, Germany) diffractometer with CuK_*α*_ radiation (*λ* = 1.5418 Å) at room temperature. The morphology of the zeolitic materials was observed by using a scanning electron microscope (SEM VEGAS 3 SBU, Tescan, Brno-Kohoutovice, Czech Republic) with an EDX detector (Quantax EDS, Bruker, Karlsruhe, Germany). Thermogravimetric analysis (TGA) was performed using a SDT O 600 (TA Instruments, New Castle, DE, USA) with temperatures ranging from 30 to 1000 °C. Total surface area, pore radius, and total pore volume were measured from the N_2_ adsorption–desorption isotherms using the Dollimore–Heal model for porosity data and the Brunauer–Emmett–Teller (BET) technique for total surface area.

### 2.3. Preparation of Silver-Exchanged Zeolite

#### 2.3.1. Zeolite Treatment with Sodium Salt

The thermally treated NZ (200 °C) was first exchanged in the Na^+^ form (NZ-Na^+^) because Na^+^ in clinoptilolite is more easily exchanged with hydrated Ag^+^ ion than the other exchangeable cations. This is due to the similar value of the ionic radius of the hydrated Na^+^ ion (3.58 Å) and that of the hydrated Ag^+^ ion (3.43 Å), while the other exchangeable cations in clinoptilolite have different values: Ca^2+^ (4.12 Å), Mg^2+^ (4.28 Å), and K^+^ (3.31 Å) [30]. To obtain the Na^+^ exchanged form (NZ-Na^+^), 240 g of NZ was treated three times with 270 mL of 0.3 M NaCl solution under reflux conditions at 100 °C for 24 h. After decantation, the zeolitic material was washed with distilled water until the Cl^−^ test with AgNO_3_ was negative. The separated solid (NZ-Na^+^) was dried at 85 °C for 6 h.

#### 2.3.2. Zeolite Treatment with AgNO_3_

The NZ-Na^+^ sample (100 g) was suspended in 0.1 M AgNO_3_ solution (1000 mL) and the pH of the solution was adjusted to 5.0. The Ag^+^ modification was performed at room temperature in the dark. To increase the sorption rate of Ag^+^ in clinoptilolite, which ensures the desorption of air from clinoptilolite particles and accelerates the diffusion of Ag^+^ ions [36], the modification was performed using an ultrasonic bath (Sonorex RK 1050, Bandelin, Berlin, Germany) at an ultrasonic frequency of 35 Hz for 16 h. The resulting solid was separated by filtration, washed several times with distilled water, and dried overnight at room temperature. The solid obtained was named NZ-Ag^+^.

### 2.4. Experimental Setup for ^222^Rn Adsorption

**Figure 1:**
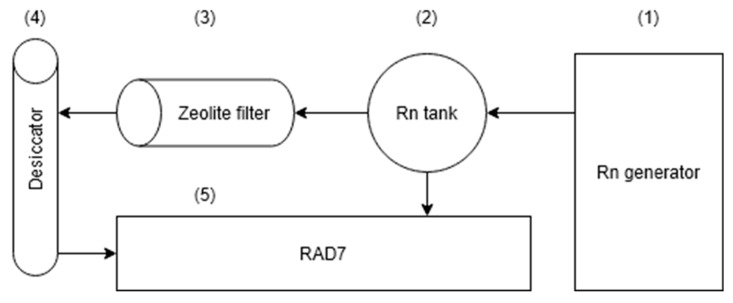
Schematic experimental setup for ^222^Rn adsorption system.

For the experimental evaluation of the ^222^Rn adsorption on the NZ and NZ-Ag^+^, a dedicated test system was considered to ensure the reproducibility of the data. As shown schematically in Figure 1, the test system was designed as a closed circuit consisting of four distinct parts: the cell containing the desiccant material (1), the radon tank (2), the adsorbent sample cell, used as a zeolite filter (3), a desiccator to remove the water vapor (4), and the RAD7 real-time radon measurement device (5). The Rn generator (1) contained sand sampled from an area near the Băiţa pit uranium deposit (Bihor County, Romania) to obtain the desired Rn concentration, which was introduced into the pre-vacuumed 2 L flask. The Rn concentration was determined by repeated 5 min measurements, while in the case of overly high concentrations, ambient air was introduced into the system. The mixing was repeated until the desired initial values were obtained. However, since a natural source of Rn is used, the resulting concentration could not be easily adjusted. Since the order of magnitude is an important factor to consider, we used three values of initial Rn concentration for each adsorbent in order to study the effect of the initial concentration on the adsorption factor.

After the concentration had stabilized, NaCl was introduced into the adsorbent sample cell system (in the tube in which the zeolite would be placed in the adsorption experiments), to investigate possible leakage of the system). A higher initial concentration of Rn was chosen for the salt experiment to create a larger concentration gradient between the closed test system and the external environment. Thus, any leakage would be more pronounced and easier to detect, and any leakage could be stopped.

In order to increase the accuracy of the determination, the following measurement protocol was considered: a 30 min measurement cycle, in which the system was pumped for 1 min and measured for 4 min. This process was repeated six times, and the value obtained represents a long-term measurement of 30 min. The measurements cover 99 cycles, the maximum number indicated by the device (2970 min).

## 3. Results and Discussion

### 3.1. Characteristics of Zeolite Samples

#### 3.1.1. Physical–Chemical and Mineralogical Characteristics of NZ and NZ-Ag^+^

The NZ composition was determined to be 65.22% SiO_2_, 12.41% Al_2_O_3_, 2.71% CaO, 2.63% K_2_O, and 1.69% of other elements. In addition, the presence of MgO, Na_2_O, Fe_2_O_3_, MnO, and LOI was identified, along with other components that collectively constituted 3.50% of the total composition. The measured Si/Al ratio was >4 and the content of alkaline cations (Na + K) was higher than that of Ca, indicating the clinoptilolite as the major component [36]. The CEC value calculated as the sum of exchangeable cations was 136.4 meq 100 g^−1^. Exchangeable Ca^2+^ was the main component of the total CEC, followed by exchangeable K^+^ and Na^+^ and Mg^2+^. Considering the total concentrations of Ca^2+^, K^+^, Na^+^, and Mg^2+^ measured in the sample after microwave acid digestion and the concentrations of exchangeable cations, it was found that Na^+^ was the most mobile cation (94% in exchangeable form), followed by Ca^2+^ (64%), K^+^ (61%) and Mg^2+^ (6.6%). The obtained NZ-Ag^+^ contained 63.79% SiO_2_, 12.33% Al_2_O_3_, 2.53% CaO, 2.71% K_2_O, 1.65% MgO, 0.76% Na_2_O, 1.33% Fe_2_O_3_, 0.03% MnO, 1.04% Ag, 10.40% LOI, and other components 3.43%.

**Figure 2:**
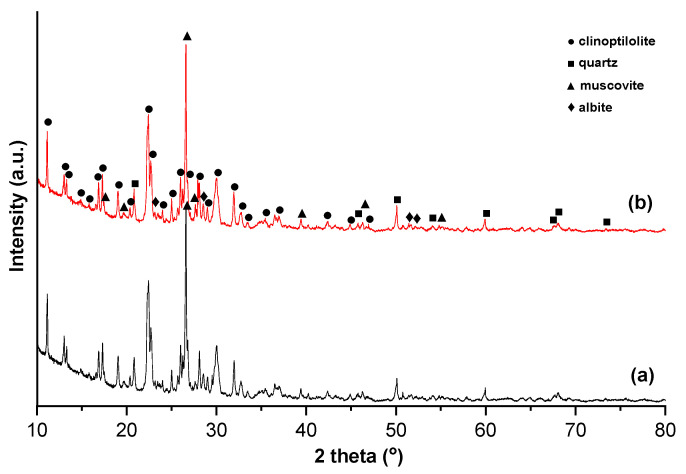
X-ray diffraction patterns of the zeolite samples: (**a**) NZ and (**b**) NZ-Ag^+^.

X-ray diffraction (XRD) analysis, displayed in Figure 2, indicates the presence of clinoptilolite as the major zeolite mineral phase.

According to XRD analysis, the zeolite samples contain clinoptilolite (PDF 00-039-1383) as the main crystalline phase, accompanied by muscovite (PDF 00-060-1516), quartz (PDF 01-077-1060), and albite (PDF 01-073-3990) (Figure 2). The hump in the region 2θ = 15–25° indicates the low amorphous content, which has been attributed to the presence of quartz and kaolinized volcanic ash tuff [37]. The Reference Intensity Ratio (RIR) method [38] used for the quantitative phase analysis indicates that the zeolite sample contains zeolites (70% clinoptilolite) accompanied by plagioclase feldspars (8%), silica polymorphs (5%), and clay minerals (17%). There is no difference in the XRD patterns of the natural and modified Ag samples, indicating that the crystal structure of the clinoptilolite was not significantly affected by the Ag modification, which is consistent with the results previously reported in other studies [39,40,41].

**Figure 3:**
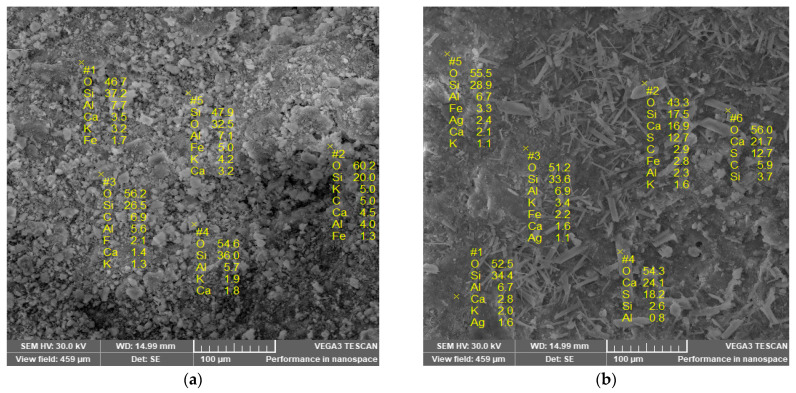
SEM images of zeolite samples: (**a**) NZ and (**b**) NZ-Ag^+^.

**Figure 4:**
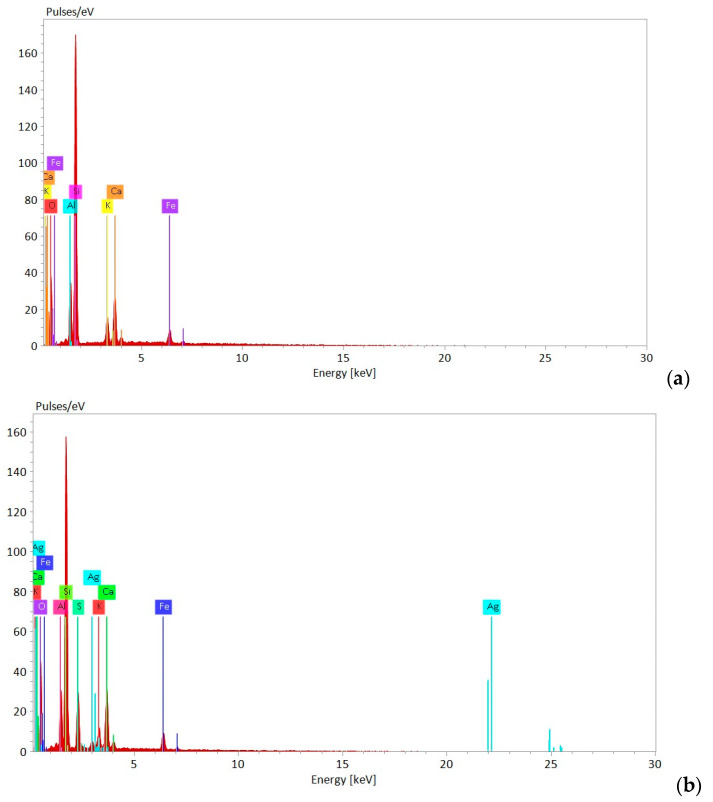
EDX Analysis of Zeolite Samples: (**a**) NZ and (**b**) NZ-Ag^+^.

The SEM images presented in Figure 3 revealed the difference between the NZ and NZ-Ag^+^ samples. The natural zeolite showed small particles grouped in different shapes such as spheres and plates as depicted in Figure 3a. The Ag exchange results in a significant transformation of the zeolite shape into plate- and cylinder-shaped particles, and a more pronounced opening of the exchanged zeolite structure, as presented in Figure 3b. Farvad et al. also reported small clustered particles for the natural zeolite that underwent significant transformation after Ag^+^ exchange and even deformation in the zeolite particles after the ion exchange process with silver nitrate and high-temperature treatment [42]. The change in the morphology of NZ when modified with Ag^+^ ions could also be due to the use of ultrasound, which accelerates the diffusion of Ag^+^ ions in the clinoptilolite channels and ensures the desorption of air from the clinoptilolite particles [33]. The EDX analysis revealed the presence of Ag in the modified zeolite (NZ-Ag^+^) as presented in Figure 4.

The EDX spectra of NZ displays the presence of elements typically found in natural zeolite, like Si, Al, O, K, Ca, Fe, and Na (Figure 4). After modification with Ag^+^, new peaks appeared for Ag, as can be observed in Figure 4b. Since the XRD pattern did not change after Ag loading (Figure 2), indicating that the zeolite framework was not changed, it can be assumed that the Ag^+^ ions appearing in the EDX spectra were simply retained on the zeolite surface.

#### 3.1.2. Physical–Chemical Characteristics of Natural Zeolite

**Figure 5:**
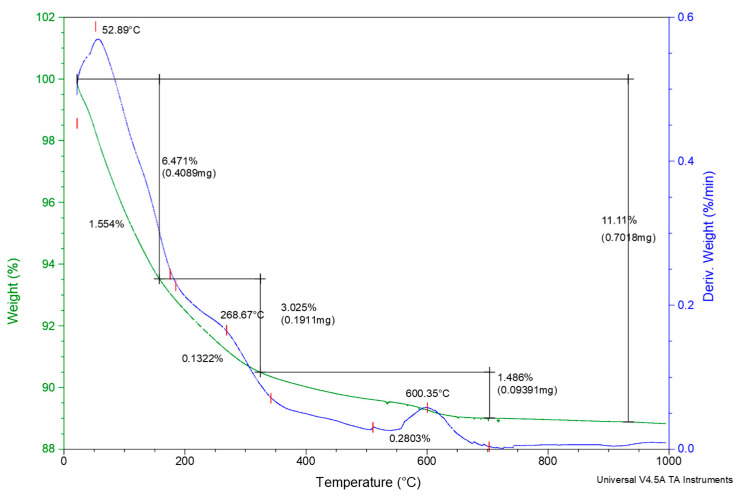
Thermal Analysis of the NZ Sample.

The thermogravimetric analysis (TGA) of the unmodified zeolite sample is presented in Figure 5.

According to the TGA, the NZ sample displays a low total weight loss of 11.11%. A weight loss of 6.74% was registered up to 150 °C and attributed to the moisture and physically adsorbed water, 3.025% at 150–350 °C was assigned to the water located within the zeolite cavities and bound to the loss of extra-framework cations, whereas 1.486% at around 600 °C in the final dehydration stage was attributed to the elimination of hydroxyl groups and isolated OH groups (structural water). The high thermal stability is characteristic for the clinoptilolite mineral, which is more stable compared to heulandite (stable up to 450–550 °C) [43].

The BET surface area of the NZ zeolite was determined to be 38 m²/g, with a corresponding pore volume of 0.059 cm^3^/g and a pore radius of 25 Å. The modification of NZ with Ag resulted in a slight decrease in the BET surface area to 33 m^2^/g, with a corresponding pore volume of 0.051 cm^3^/g. This represents a decrease in the BET surface area of about 13%, and a decrease in the pore volume of about 14%, which means less space for gas adsorption. The decrease could be explained by the incorporation of Ag^+^ into the zeolite pores after the chemical modification process. A decrease in BET surface area and total pore volume after incorporation of Ag^+^ into zeolite was also reported by Kulawong et al. [44] and Akhigbe et al. [39]. Faryad et al. [42] also observed a decrease in BET surface area and porosity of mordenite–clinoptilolite zeolite after the modification with AgSO_4_ or AgNO_3_. A possible explanation could be the blocking of the natural zeolite micropores by the larger volume Ag^+^ ions, leading to the restriction of the available area for N_2_ adsorption in the BET measurement process.

### 3.2. Radon Adsorption Experiments

#### 3.2.1. System Leak Tests

The Rn concentration in the system follows the decay law, which overlaps the radon leakage from the system and, respectively, with the absorption on the zeolite when the absorbent cartridge is inserted in the system presented in Figure 1. When investigating the leakage from the system, we addressed the difference between the theoretical value of the decay constant and the value obtained by fitting the values to these equations:

(1) $${A=A}_{0}\cdot e^{\left(b\right)\cdot t}$$

(2) b=λ+β+ε

with *A*-activity ^222^Rn, *A_0_*_-_activity at the beginning of the experiment, λ the decay constant of ^222^Rn (1.25 10^−4^ min^−1^), β-absorbance constant on zeolite, ε-leakage constant (preferred value 0) and *b*-total attenuation constant (obtained from the splitting).

**Figure 6:**
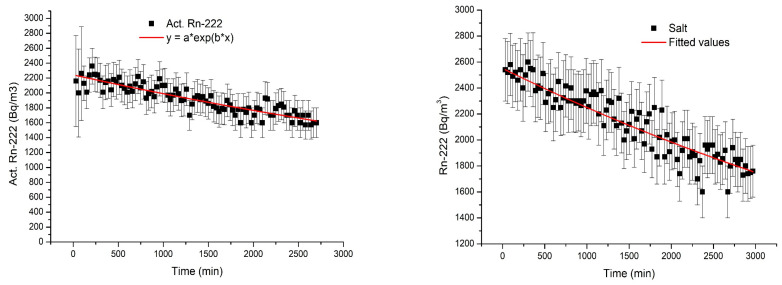
System leakage over the entire measurement process.

Figure 6 shows the results of the system leakage test presented over the entire measurement process. The ^222^Rn as a function of time inside the Rn tank was determined using the RAD7 alpha-spectrometer.

Considering that the average system leakage over the entire measurement process of 0.08 10^−4^ min^−1^ is comparable to the individual measurement errors, it can be considered negligible. Error bars shown for each individual measurement indicate the statistical errors 2σ that account for the toxic radioactive disintegration of ^222^Rn combined with the measurement uncertainty of the RAD7 equipment.

#### 3.2.2. Radon Adsorption by NZ

**Figure 7:**
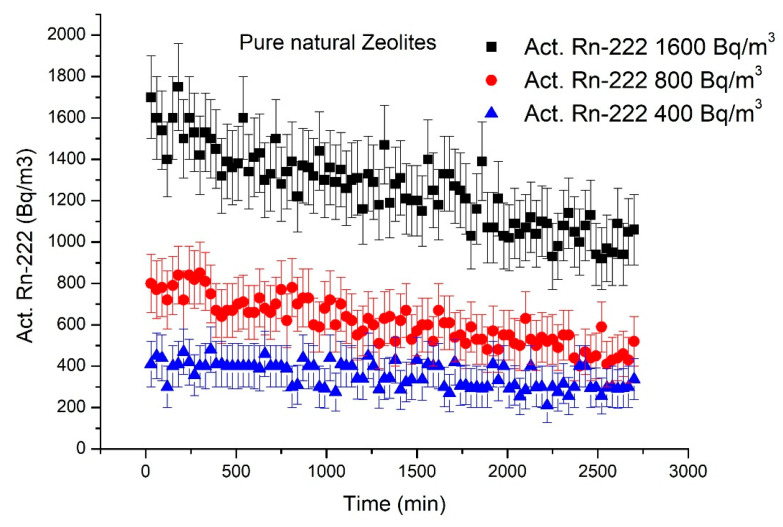
Evolution of ^222^Rn activity in the system over 99 cycles (2970 min) measurement period recorded for three levels of initial concentration (1600 Bq/m^3^—black line; 800 Bq/m^3^—red line; 400 Bq/m^3^—blue line) using NZ as filtering material. Error bars showing the statistical errors 2σ that account for the poison radioactive disintegration of ^222^Rn combined with the measurement uncertainty of RAD7 equipment are presented for each single measurement.

The clinoptilolite-type zeolite thermally treated at a temperature of 200 °C and with particle sizes of 0.5–1.0 mm was placed in the cartridge and tested as a ^222^Rn adsorbent. The data points in Figure 7 show the ^222^Rn activity measured over a 2970 min recording period, starting from three initial radiation levels: 1600, 800, and 400 Bq/m^3^, respectively.

A decrease in the ^222^Rn activity in the system was registered when the unmodified NZ was used as filter material (Figure 7). In the case of an initial ^222^Rn activity of ~1600 Bq/m^3^, the decrease was observed up to a measurement period of 2000 min, where the ^222^Rn activity remained almost constant at ~1050 Bq/m^3^. In the second experiment, in which the initial ^222^Rn activity was ~800 Bq/m^3^, the decrease was also observed up to a measurement period of 2000 min, to a value of ~520 Bq/m^3^. When the initial ^222^Rn activity introduced into the system was ~400 Bq/m^3^, a very slow decreasing trend was observed, also up to a measurement time of 2000 min, to a value of ~280 Bq/m^3^. Thus, the adsorption capacity of natural zeolite is higher in environments with a higher ^222^Rn activity. Considering the mass of adsorbent material (31.7 g) and the difference between the initial activity and the value at which this remains constant, the ^222^Rn activity (*Q*) adsorbed per unit mass of sorbent material can be estimated. Using the *Q* value and the activity of ^222^Rn in air (*C*) (Bq/m^3^), an adsorption coefficient (*k*) can be calculated using Equation (3) [19]:

(3) k=QC

The *Q* values estimated for the three adsorption experiments were 17.35, 8.83, and 3.79 Bq/g for initial ^222^Rn activities of ~1600 Bq/m^3^, 800 Bq/m^3^, and 400 Bq/m^3^, respectively. The corresponding adsorption coefficients *k* (m^3^/g) were 0.0108, 0.0110, and 0.0095, values that are very close to each other regardless of the initial ^222^Rn concentration. These values are two orders of magnitude higher than those reported by Bikit et al. [19] for unmodified natural zeolite, (0.038 m^3^kg^−1^–0.11 m^3^kg^−1^). However, in that case the zeolite was only exposed in a glass chamber with a volume of 5.4 L, whereas in our experiments the contaminated air was circulated for 2970 min through a filter containing zeolite. Our data show that natural zeolite can reduce ^222^Rn activity and may be suitable for reducing radioactivity in highly contaminated environments.

#### 3.2.3. Radon Adsorption by NZ-Ag^+^

**Figure 8:**
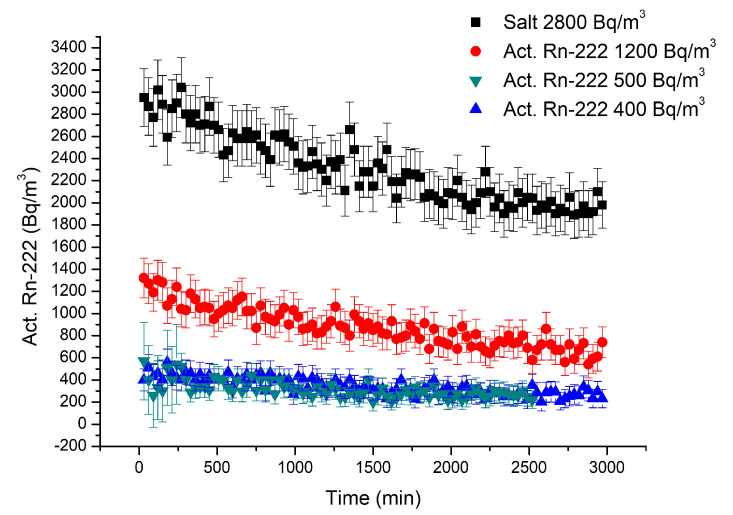
Time dependence of ^222^Rn concentration in the system over 99 cycles’ (2970 min) measurement period recorded for three levels of initial concentration (1200 Bq/m^3^—red line; 500 Bq/m^3^—green line; 400 Bq/m^3^—blue line) using NZ-Ag^+^ as filtering material and salt as inert material (black line). Error bars shown for each individual measurement indicate the statistical errors 2σ that account for the toxic radioactive disintegration of ^222^Rn combined with the measurement uncertainty of the RAD7 equipment.

The silver-exchanged NZ with particle sizes of 0.5–1.0 mm introduced into the cartridge and tested as a ^222^Rn adsorbent provided the adsorption data presented in Figure 8. The ^222^Rn activity was also measured over a recording period of 2970 min, for 99 measurement cycles. In this experiment, the initial ^222^Rn activities were 1200 Bq/m^3^, 500 Bq/m^3^, and 400 Bq/m^3^, respectively.

According to the data presented in Figure 8, when NZ-Ag^+^ was used as a filter material, a decreasing trend of ^222^Rn activity in the system was also obtained. As in the case of NZ, the decrease stopped after about 2000 min of adsorption. When the initial ^222^Rn activity was ~1200 Bq/m^3^, a constant value was obtained at ~70 Bq/m^3^; and when the initial ^222^Rn activity was ~500 Bq/m^3^, the decrease stopped at ~270 Bq/m^3^; whereas when the initial ^222^Rn activity was ~400 Bq/m^3^, the obtained constant value was ~230 Bq/m^3^. Taking into account the mass of the adsorbent material (31.7 g) and the difference between the initial activity and the value at which it remains constant, the *Q* values in the case of NZ-Ag^+^ were estimated to be 22.08, 8.52, and 7.26 Bq/g for initial ^222^Rn activities of ~1200, 500, and 400 Bq/m^3^, respectively. The corresponding adsorption coefficients *k* (m^3^/g) in this case were 0.0184, 0.0170, and 0.0181, with values also very close to each other regardless of the initial ^222^Rn activity. Thus, in all three experiments, the loading of NZ with Ag^+^ ions resulted in an increase of the adsorption coefficients by about 40%, showing that the silver improves the Rn capture by forming weak interactions with Rn atoms. Nevertheless, future studies are necessary to observe if the Ag^+^ loading level correlates with the adsorption efficiency, if higher Ag content on natural zeolite leads to better performance, or if there is an optimal range.

#### 3.2.4. ^222^Rn Removal Performance of Each Adsorbent

To quantify the ^222^Rn removal performance of NZ and NZ-Ag^+^, the values for the adsorption factors (*ε*) were calculated and are presented in Table 1.

The zeolite absorption factors can also be expressed in percentages and the corresponding values are given in Table 2 for one hour of Rn gas recirculation (pump flow rate of 1 L min^−1^). The percentage absorption per gram of zeolite was calculated for a mass of 31.7 g of absorbent, used to fill the adsorption cell.

As summarized in Table 1 and Table 2, the ^222^Rn removal of both NZ and NZ-Ag^+^ increased with time and reached their maximum values at about 2000 min of air circulation in the system. Considering that the gases are trapped into the pores of zeolites, the reduction of the BET surface area and the pore volume may hinder the Rn adsorption. Despite this issue, the retention capacity of NZ-Ag^+^ is superior to that of NZ, probably due to the interaction between Rn and Ag^+^ ions. In addition, the incorporation of Ag^+^ ions changes the electrostatic potential of the zeolite surface it occurs upon, resulting in a greater affinity for noble gases [45].

From the data presented in Table 2, it can be observed that the maximum retention rate of the silver-exchanged zeolite was found to be 50% higher than that of the natural zeolite. By increasing the amount of absorbing zeolite, about 56% of the initial ^222^Rn value can be retained when using NZ-Ag^+^ as the adsorbent. The increase of ^222^Rn adsorption by Ag-exchanged zeolite can be explained by the interaction between the noble gas atoms and Ag^+^ ions loaded on the zeolite surface [28,46] because the *d* orbitals of Ag^+^ ions favor a *σ* donation between the *5s* orbital of silver and the *5p* orbital of the noble gas [22,47]. Other authors considered that a significant change of the electrostatic potential takes place on the surface of the zeolite after its modification with Ag nanoparticles, which improves the affinity to noble gas, including Rn [25].

The improvement of the adsorption characteristics of zeolites by Ag^+^ modification has also been reported previously. In this regard, Heinitz et al. [25] reported significantly higher adsorption coefficients for Rn when three Ag-modified synthetic zeolites were tested, ranging from ~30 to 3000 m^3^/kg, compared to those of the activated carbons NuclearCarb 207C and CarboAc, whose adsorption coefficients were 5.6 and 7.2 m^3^/kg, respectively. Veselska et al. [26] reported good results for adsorption of Rn traces in low background experiments by silver-exchanged zeolites. Mortazavi et al. [48] reported that natural zeolite and bentonite reduced indoor Rn concentrations. The efficiency of Rn removal was observed by the reduction of airborne Rn activity. He et al. [21] proved that zeolite, barite, iron oxide, high alumina cement can effectively adsorb the release of Rn from fly ash. Among these adsorbents, zeolite was found to be the most efficient. The composite filters containing zeolite in percentages of 5, 10, and 15% provided about 70% removal of the radioactivity [20]. The reported adsorption coefficients of 15 m^3^/kg for Ag-13X, 7 m^3^/kg for Ag-ZSM-5, and 1400 m^3^/kg for Ag-ETS-10 indicated that not only the adsorbent but also the experimental conditions affect the adsorption behavior [25].

Overall, our results indicate that the chemical modification of natural zeolite with Ag^+^ ions increases its ^222^Rn removal capacity and can be used to decrease Rn activity in the air, especially considering the wide availability of natural zeolite, and the simplicity of the proposed methodology for its chemical modification. These results are in agreement with previous studies carried out on synthetic zeolites reporting the improvement of the adsorption characteristics after their modification with silver [24,25,26,29,49]. Nevertheless, future studies are necessary to find correlations between the Ag^+^ loading level and the adsorption efficiency of the material. An important issue is the management of the adsorbent material after the adsorption and its reusability. In this regard, Hedström [24] pointed out a possible regeneration of the zeolite by heating at 250 °C for 12 h.

## 4. Conclusions

In this study, we developed a methodology for the removal of ^222^Rn from air by its retention on an adsorbing material. We reported for the first time in the literature the use of silver-exchanged natural zeolite rich in clinoptilolite for this purpose. Natural zeolite obtained from Racos quarry, Romania, with particle sizes of 0.5–1.0 mm, was thermally treated at 200 °C and further chemically modified by loading with silver ions to enhance its adsorption characteristics. The natural zeolite and its modified form were characterized using ICP-OES, SEM-EDX, XRD, TGA, and BET techniques to assess their characteristics. A special test system was designed to monitor the Rn removal by the adsorbents and to assure the reproducibility of the data. According to ICP-OES, the Ag content in the modified zeolite was about 1%, while the EDX spectra confirmed the presence of Ag in the exchanged zeolite. The XRD patterns showed that the crystal structure of the clinoptilolite was not significantly affected by the modification of the zeolite samples. The ^222^Rn removal of both NZ and NZ-Ag^+^ increased with time, reaching maximum values at about 2000 min of air circulation in the system. An improvement in the maximum retention rate of the silver-exchanged zeolite was found to be 50% higher than that of the unmodified zeolite. Further investigations are still required to fully understand the influence of Ag loading on zeolites towards increasing Rn adsorption, since the modified natural zeolite may be a promising filter material for radon removal with applications in indoor air purification and minimization of human radon inhalation.

## Figures and Tables

**Table 1:** Adsorption factors (ε) for ^222^Rn on NZ, and NZ-Ag^+^.

Tests	NZ (×10^−4^ min^−1^)	Tests	NZ-Ag^+^ (×10^−4^ min^−1^)
Test 1 (1600 Bq/m^3^)	0.484	Test 1 (1200 Bq/m^3^)	0.855
Test 2 (800 Bq/m^3^)	0.846	Test 2 (500 Bq/m^3^)	0.836
Test 3 (400 Bq/m^3^)	0.160	Test 3 (400 Bq/m^3^)	0.549
Mean	0.496	Mean	0.746
Geometrical mean	0.403	Geometrical mean	0.732

**Table 2:** Adsorption factors for ^222^Rn on NZ and NZ-Ag^+^ expressed as percentages.

	NZ	NZ-Ag^+^
%Ads/h	1.192	1.792
%Ads/H·g	0.037	0.056

## Data Availability

The original contributions presented in this study are included in the article. Further inquiries can be directed to the corresponding authors.
